# High-Level Expression, Purification and Initial Characterization of Recombinant *Arabidopsis* Histidine Kinase AHK1

**DOI:** 10.3390/plants9030304

**Published:** 2020-03-01

**Authors:** Alexander Hofmann, Sophia Müller, Thomas Drechsler, Mareike Berleth, Katharina Caesar, Leander Rohr, Klaus Harter, Georg Groth

**Affiliations:** 1 Institute of Biochemical Plant Physiology, Heinrich Heine University, Düsseldorf 40225, Germany; a.hofmann@hhu.de (A.H.); somue106@uni-duesseldorf.de (S.M.); mareike.berleth@hhu.de (M.B.); 2 Center for Plant Molecular Biology (ZMBP), Eberhard Karls Universität Tübingen, Tübingen 72076, Germany; thomas.drechsler@zmbp.uni-tuebingen.de (T.D.); katharina.caesar@mwk.bwl.de (K.C.); leander.rohr@zmbp.uni-tuebingen.de (L.R.); klaus.harter@zmbp.uni-tuebingen.de (K.H.)

**Keywords:** histidine kinase, osmosensing, membrane protein purification, phosphorelay, protein-protein interaction

## Abstract

Plants employ a number of phosphorylation cascades in response to a wide range of environmental stimuli. Previous studies in *Arabidopsis* and yeast indicate that histidine kinase AHK1 is a positive regulator of drought and osmotic stress responses. Based on these studies AHK1 was proposed a plant osmosensor, although the molecular basis of plant osmosensing still remains unknown. To understand the molecular role and signaling mechanism of AHK1 in osmotic stress, we have expressed and purified full-length AHK1 from *Arabidopsis* in a bacterial host to allow for studies on the isolated transmembrane receptor. Purification of the recombinant protein solubilized from the host membranes was achieved in a single step by metal-affinity chromatography. Analysis of the purified AHK1 by SDS-polyacrylamide gel electrophoresis (SDS-PAGE) and immunoblotting show a single band indicating that the preparation is highly pure and devoid of contaminants or degradation products. In addition, gel filtration experiments indicate that the preparation is homogenous and monodisperse. Finally, CD-spectroscopy, phosphorylation activity, dimerization studies, and protein–protein interaction with plant phosphorylation targeting AHP2 demonstrate that the purified protein is functionally folded and acts as phospho-His or phospho-Asp phosphatase. Hence, the expression and purification of recombinant AHK1 reported here provide a basis for further detailed functional and structural studies of the receptor, which might help to understand plant osmosensing and osmosignaling on the molecular level.

## 1. Introduction

Plants are sessile organisms that have to adapt to fluctuating environmental conditions during their life cycle. To sense, respond, and adapt to a wide range of environments, stressors, and growth conditions, plants employ phosphorylation cascades that are already found in their basic genetic set-up in archaea, bacteria and fungi [1]. However, in contrast to the typical prokaryotic two-component phosphotransfer systems (TCS) involving a single His-Asp phosphotransfer process from a soluble or membrane-bound sensor histidine kinase to the receiver domain of a cognate response regulator protein, plants employ multi-step His-Asp phosphotransfer cascades (phosphorelays) encompassing hybrid-type sensors that carry kinase and receiver domains within the same molecule, His phosphotransfer proteins, and response regulator proteins [2].

Altogether the genome of *Arabidopsis thaliana* contains 11 genes encoding histidine kinases (HK) [3,4]. Sequence analysis suggests that all members of the HK family have a modular basic structure consisting of an N-terminal transmembrane sensor domain followed by a catalytic transmitter domain and a receiver domain at the C-terminus. Biochemical, biophysical, and cellular studies show that the receptors form dimers and higher-molecular-weight oligomers at the membrane in their functional state [5,6,7,8,9,10,11]. Further studies demonstrate that five members of the *Arabidopsis* HK family encode receptors sensing the plant hormone ethylene (ETR1, ERS1, ETR2, ERS2, and EIN4) [12,13,14], whereas the other six encode non-ethylene receptor kinases (AHK1, AHK2, AHK3, CRE1/AHK4, CKI1, and CKI2/AHK5). Three of the non-ethylene receptors (AHK2, AHK3, and CRE1) have been identified as receptors for the plant hormone cytokinin [15,16,17]. The residual three HK receptors (AHK1, CKI1, and AHK5) show neither ethylene nor cytokinin-related activity and have been attributed to a variety of plant processes including osmoregulation [18], megagametogenesis [19], salt sensitivity [20], or stomatal responses [21], although the molecular trigger or ligand stimulating these activities is not known for all of these processes yet.

While the overall architecture is conserved for all members of the *Arabidopsis* HK family, some of them lack histidine kinase activity due to a degenerate catalytic domain (ETR2, ERS2, and EIN4) or miss a receiver domain at their C-terminus (ERS1 and ERS2). Consequently, these isoforms cannot participate in a canonical phosphorelay, but may contribute to phosphotransfer to downstream proteins via the formation of heterodimers with other fully functional members of the *Arabidopsis* HK family. Still, the most striking difference in the architecture of the *Arabidopsis* HK family is found in their sensor domain. For the ethylene receptor kinases this domain is transmembrane and contains a bound copper cofactor coordinated by three (ETR1 and ERS1) or four (ETR2, ERS2, and EIN4) transmembrane α-helices, whereas for the non-ethylene receptor kinases involved in cytokinin signaling the ligand binding domain (CHASE) is located in a loop connecting their two (AHK4), three (AHK3) or four (AHK2) transmembrane spans. Similarly, to the AHK4 cytokinin receptor prototype two of the non-plant hormone receptor HKs also contain two putative transmembrane helices (CKI1 and AHK1) in their N-terminal sensor domain, whereas sequence analysis of AHK5 indicates no obvious transmembrane element in this receptor kinase. Although AHK1 has been attributed to osmosensing [18], the molecular basis of plant osmosensing is still unknown.

Biochemical and biophysical studies on purified receptors or their subdomains have substantially expanded our molecular understanding of ethylene and cytokinin sensing and signaling in the past [22,23,24,25,26,27]. Due to their low abundance in their natural host, recombinant production of the related proteins was a critical prerequisite for these studies. Hence, in order to expand our knowledge on the putative osmosensor AHK1, we have expressed this non-plant hormone transmembrane receptor HK in a bacterial system and purified the full-length recombinant protein from the bacterial host to homogeneity. Functional folding of the purified detergent-solubilized AHK1was verified by CD-spectroscopy, phosphorylation activity assays, and protein interaction studies with downstream *Arabidopsis* histidine-containing phosphotransfer proteins (AHPs). Consequently, the high-level expression and efficient purification of recombinant *Arabidopsis* osmosensor histidine kinase AHK1 reported in this work provide a critical milestone for further mechanistic and structural studies on a plant osmosensor candidate which might help to resolve plant osmoregulation and signaling on the molecular level.

## 2. Results 

### 2.1. Heterologous Expression and Purification of AHK1 Sensor Kinase 

**Figure 1:**
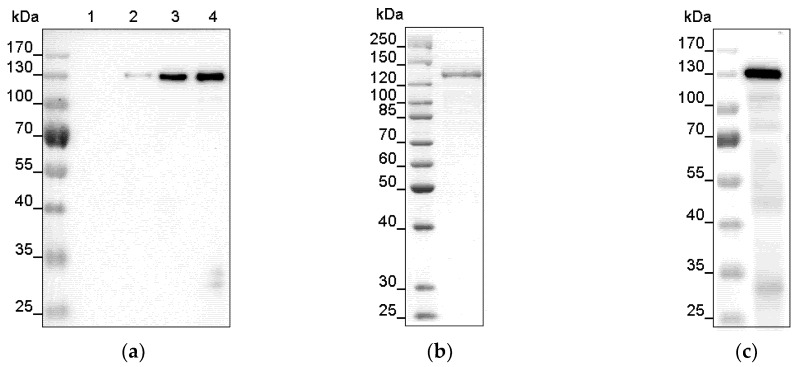
Expression and purification of recombinant His-tagged AHK1. (**a**) Expression of *Arabidopsis* histidine kinase 1 (AHK1) in *E. coli* BL21 (DE3) containing the pRARE plasmid was checked on 10% SDS-polyacrylamide gel electrophoresis (SDS–PAGE). Samples from the expression were taken upon induction with isopropyl β-d-1-thiogalactopyranoside (IPTG) (lane 1) and after 2, 4, and 5 h, respectively (lanes 2–4) and analyzed by Western blotting using anti-His Tag antibodies. (**b**) AHK1 purified by nickel-nitrilotriacetic acid (Ni-NTA) affinity chromatography. Left lane, protein marker; right lane, 1 µg of purified protein. Protein bands were visualized by Coomassie staining. The apparent molecular mass of about 130 kDa of the purified protein corresponds to the theoretical molecular mass of AHK1. (**c**) Purity and identity of the recombinant AHK1 was confirmed by Western blotting using anti-His Tag antibodies.

In this study we successfully expressed His-tagged recombinant full-length *Arabidopsis* histidine kinase AHK1 in the bacterial host *E. coli* BL21 (DE3). Systematic screening of culture conditions identified a growth temperature of 16 °C after induction as the most suitable to obtain sufficient amounts of the recombinant plant histidine kinase at maximal stability. Further experiments revealed that translational errors leading to protein aggregation can be minimized by the addition of plasmid pRARE which provides rare codon tRNAs during bacterial expression. Extra addition of 2% (*v*/*v*) ethanol in the expression culture further reduced the tendency to form inclusion bodies. With these conditions and in culture medium Terrific broth (TB) (Figure S1), high protein levels with minimal contaminations were obtained 5 h after induction (Figure 1a). The observed small contaminations at 30 kDa may be attributed to protein degradation or incomplete protein translation. Upon longer expression times these contaminations increased relatively to the target protein rendering these conditions less favorable for recombinant AHK1 production in the bacterial host.

In subsequent systematic detergent screens (Figures S2 and S3, Table S1), 1% (*w*/*v*) FosCholine-16 was identified as most efficient in solubilizing the recombinant plant histidine kinase from the bacterial membranes. Whole cell *E. coli* lysate solubilized this way was clarified by centrifugation at 230,000× g and applied to nickel-nitrilotriacetic acid (Ni-NTA) immobilized metal affinity chromatography (IMAC). Nonspecifically bound proteins were removed by washing the affinity resin with 50 mM imidazole. Highly pure recombinant AHK1 was eluted at 100 mM imidazole. Coomassie staining (Figure 1b) and Western blot analysis (Figure 1c) revealed a dominant single band at approximately 130 kDa which correlated well to the sequence-based theoretical molecular mass of recombinant AHK1 of 138 kDa. While Coomassie staining of the purified sample showed no obvious signs of contaminations, some faint background bands become visible in the Western blot, suggesting that minor protein degradation may have occurred before or during chromatographic purification in the presence of 0.015% (*w*/*v*) FosCholine-16. However, due to their low intensities in comparison to the band intensity of the full-length AHK1 membrane protein these background contaminants can be neglected.

### 2.2. Size Exclusion Chromatography of the Recombinant AHK1 Osmosensor

**Figure 2:**
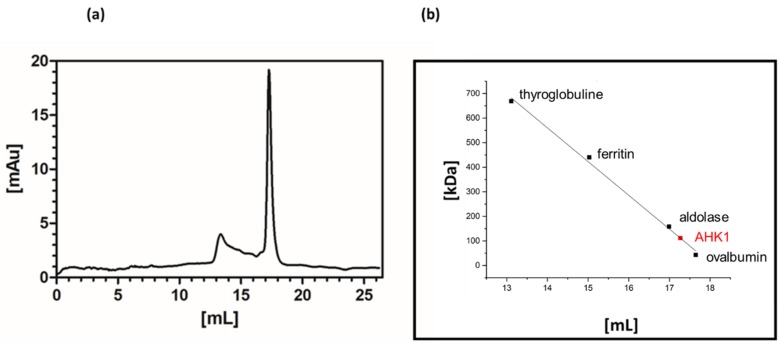
Size exclusion chromatogram of the recombinant AHK1 osmosensor (**a**). Experiments were performed on a Superose 10/300 column in 50 mM Tris, 300 mM NaCl, 10% (*w*/*v*) glycerol, 2.5 mM DTT, 0.015% (*w*/*v*) FosCholine16, 0.002% (*w*/*v*) phenylmethylsulfonyl fluoride (PMSF) at pH 7.6 with 100 µL of purified AHK1. The molecular weight of the AHK1 osmosensor was calculated based on the slope of the calibration curve obtained with standard proteins. The distinct elution peak observed at 17.27 mL corresponds to a molecular weight of 111 kDa, indicating that AHK1 was isolated in the monomeric state. Calibration curve (**b**) derived from the four elution volumes of thyroglobuline, ferritin, aldolase, and ovalbumin with the elution volume of AHK1 (17.27 mL) indicated in red.

To evaluate homgeneity and monodispersity of the recombinantly-produced osmosensor we performed a size exclusion chromatography (SEC). The SEC profile shown in Figure 2a shows a distinct major peak at 17.27 mL elution volume. Peak shape and narrow distribution indicate monodispersity and homogeneity of the purified AHK1 sensor kinase. The small minor peaks observed at lower elution volume may be attributed to larger micelles or aggregates. In order to relate the elution volume on the gel filtration column to the molecular mass of the purified full-length AHK1 sensor kinase, a number of protein standards (thyroglobuline 669 kDa, ferritin 440 kDa, aldolase 158 kDa, ovalbumin 43 kDa) were applied to the gel filtration column at the same buffer conditions. Based on the calibration with these proteins (see Figure 2b) the main peak of the elution profile corresponded to a molecular mass of 111 kDa. Assuming that the overall fold of the recombinant AHK1 resembles the globular form of the protein standards in the presence of 0.015% (*w*/*v*) FosCholine-16 and taking into account the molecular mass of the FosCholine micelle, the molecular mass of 111 kDa calculated from the SEC experiments implies that the purified recombinant AHK1 osmosensor was isolated in its monomeric form. 

### 2.3. Circular Dichroism of Purified Recombinant AHK1 Osmosensor 

**Figure 3:**
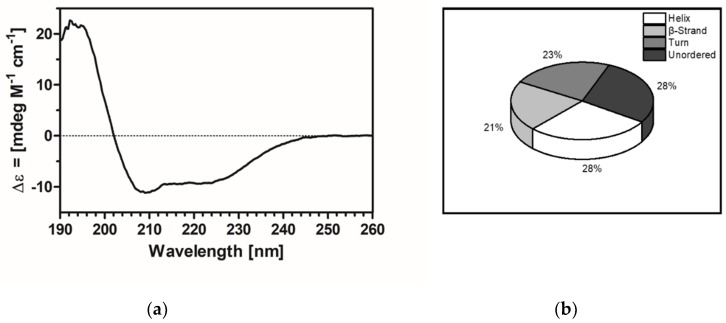
Circular dichroism of recombinant AHK1. (**a**) Circular dichroism spectrum of recombinant AHK1 shows minima at 208–210 nm and around 220 nm which are typical for α-helical structures. The spectrum has no significant negative band below 200 nm which is indicative for unordered polypeptide chains. (**b**) The secondary structure content derived from spectrum deconvolution specifies that 72% of AHK1 are present in a defined secondary structure and adopt either an α-helical, β-sheet or β-turn fold.

Purified AHK1 was analysed by circular dichroism (CD) spectroscopy to demonstrate functional folding of the recombinant protein and to exclude that heterologous expression and detergent solubilization have resulted in improperly-folded or partially-unfolded protein. The CD spectrum obtained on this sample (Figure 3a) shows minima at 205 nm and around 220 nm. Together with the maximum at 195 nm and the observed positive absorbance at wavelengths below 200 nm these minima are characteristic for a well-folded highly α-helical protein [28,29]. For a detailed analysis of secondary structure content, the CD spectrum was deconvoluted with three different algorithms (SELCON3, CONTINLL, and CDSSTR [30]) and the SMP56 membrane protein reference set (Table 1). All three algorithms gave similar results which are summarized in Table 1. However, as no experimental structural data are available for AHK1 yet, secondary structure contents obtained from CD measurements were compared to sequence-based *in-silico* secondary structures predicted by GOR4 [31] and SOPMA [32]. Data from spectroscopic analysis (Figure 3b) and *in silico* predictions (Table 1) agree well, with GOR4 resembling precisely the experimental data for the helical content. They specify that most of the recombinant protein adopts a well-defined secondary structure with only 25% of the sequence existing in an unstructured conformation which might represent the flexible loops connecting the well-defined transmembrane sensor, and catalytic topological domains. Thus, the purified recombinant AHK1 is likely to reflect the native fold and conformation of the plant histidine kinase. 

### 2.4. Dimerization Studies of AHK1 Sensor Kinase

**Figure 4:**
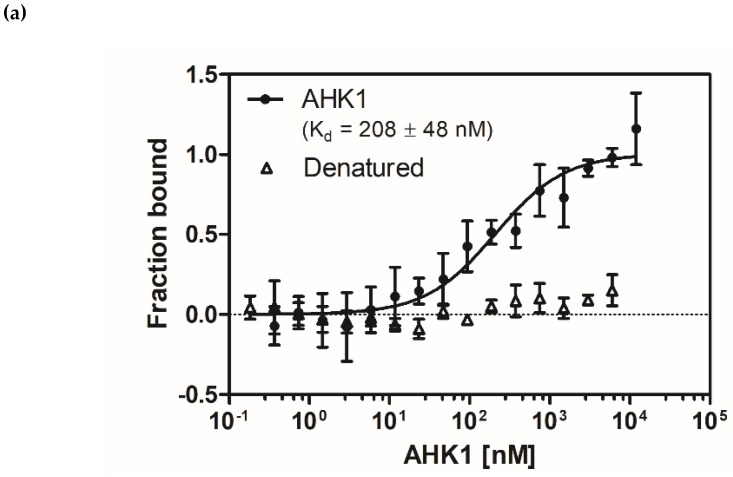

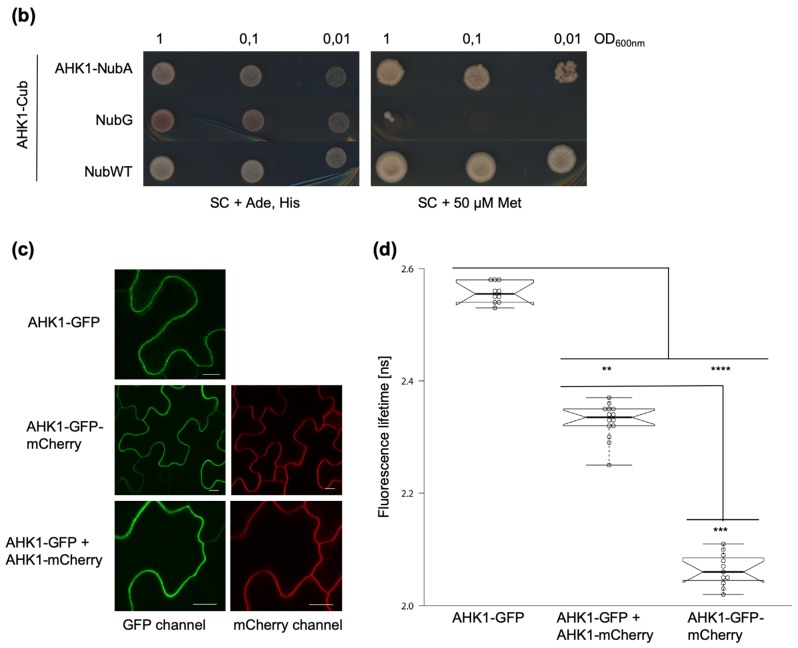
AHK1 forms homodimers in vitro and in vivo. (**a**) Microscale thermophoresis (MST) interaction studies of fluorescently labeled AHK1 sensor kinase with unlabeled AHK1. Relative changes observed in thermophoresis reflecting binding of the monomers (●) in the functional dimer were fitted according to a one-binding-site model. The related binding curve indicates a K_d_ value of 208 +/− 48 nM for AHK1-AHK1 homodimer formation. Data for chemically denatured AHK1 (△) shows no changes in thermophoresis. Data represent the mean of three independent measurements +/− standard deviation. (**b**) Yeast mating-based split-ubiquitin system (mbSUS) assay with AHK1-Cub and the indicated Nub fusion proteins. Yeast cells transfected with the corresponding expression constructs were grown at different OD_600nm_ and 30 °C on either non-interaction-selecting medium (SC + Ade, His, left) for 1 day or interaction-selecting medium (SC + 50 µM Met, right) for 4 days. (**c**) Co-localization of AHK1-GFP and AHK1-mCherry in the plasma membrane of transiently-transformed *N. benthamiana* epidermal leaf cells. Confocal images were taken 3 days after *Agrobacterium* transformation; bars: 10 µm. (**d**) FRET-FLIM analysis of the AHK1-GFP/AHK1-mCherry association in *N. benthamiana* epidermal leaf cells 3 days after Agrobacterium infection. The AHK1-GFP fusion serves as donor-only control and AHK1-GFP-mCherry fusion as positive control. Center lines in the boxplot show the medians; box limits indicate the 25th and 75th percentiles as determined by R software; the whiskers extend to minimum and maximum values. The details of the statistical evaluation are provided in Material and Methods; asterisks indicate significant differences (**: *p* ˂ 0.02; ***: *p* ˂ 0.01; ****: *p* ˂ 0.001).

Dimerization of full length AHK1 was analyzed by microscale thermophoresis (MST) measurements. Changes in initial fluorescence were detected upon addition of increasing concentrations of unlabeled full length AHK1 (0.4–12 µM) to 40 nM AHK1 labeled with the fluorophore AlexaFluor488-NHS. The resulting binding curve (Figure 4a) reflects tight binding of the monomers with an apparent dissociation constant (K_d_) of 208 +/− 48 nM. The observed high affinity binding between AHK1 monomers resulting in the formation of the dimer present at physiological conditions further supports *in vitro* functional folding of the expressed and purified full-length AHK1 osmosensor. The negative control with chemically denatured AHK1 showed no interaction as expected (see Figure 4a). To corroborate the in vitro results *in vivo*, we performed a mating-based split-ubiquitin system (mbSUS) assay in yeast [33] and Förster resonance energy transfer (FRET)-fluorescence lifetime imaging microscopy (FLIM) study in transiently-transformed *Nicotiana benthamiana* epidermal leaf cells [34]. For the mbSUS assay, full-length AHK1 and control proteins were expressed as Nub or Cub fusion in yeast. After mating, the presence of the plasmids was verified by growth of yeast cells on synthetic complete (SC) medium containing adenine and histidine (SC + Ade, His), whereas the putative interaction of AHK1 was assayed by growth on SC medium containing 50 µM Met, which enhances the stringency of the interaction test (Figure 4b). AHK1-Cub together with the negative control NubG (Nub mutant to avoid reassembling) did not show complementation of yeast growth, whilst the growth in the presence of the positive control NubWT (wild type Nub) proved the effectiveness of the assay. A clear complementation of yeast growth was observed in the presence of AHK1-Cub and AHK1-NubA (Figure 4b), demonstrating AHK1 homomerization *in vivo*. To verify the results of the MST and mbSUS studies, we performed a (FLIM) analysis. AHK1-GFP was transiently expressed in tobacco (*Nicotiana benthamiana*) epidermal leaf cells either alone (negative control) or with AHK1-mCherry. The AHK1-GFP-mCherry served as positive control. All fusion proteins (co-)localized to the cellular periphery suggesting that AHK1 is a plasma membrane-resident protein in plant cells (Figure 4c). In the presence of AHK1-mCherry the fluorescence lifetime of AHK1-GFP decreased significantly indicating energy transfer between the fluorophores and, thus, very close spatial proximity/homomerization of the AHK1 monomers (Figure 4d). The AHK1-GFP-mCherry positive control exhibited the expected very strong reduction of the fluorescence lifetime, as the donor and acceptor fluorophores were directly linked in the fusion protein (Figure 4d).

### 2.5. Heterologous Expression and Purification of the AHP2 Transfer Protein

**Figure 5:**
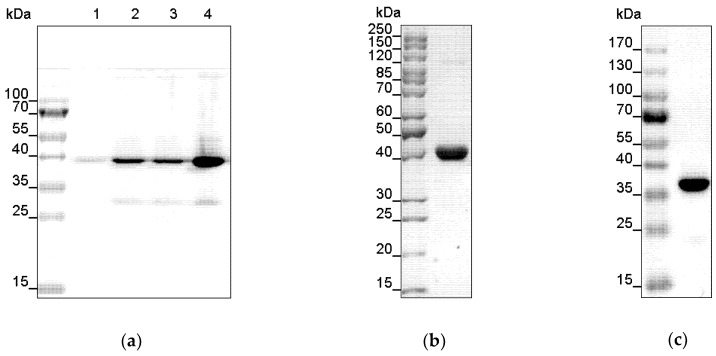
Expression and purification of recombinant GST-tagged AHP2. (**a**) Expression of GST-fusion protein of *Arabidopsis* histidine phosphotransfer protein 2 (AHP2) in *E. coli* BL21 (DE3) Gold cells was analyzed by SDS-polyacrylamide gel electrophoresis (SDS-PAGE) and subsequent Western blotting. Protein expression was monitored upon induction with IPTG (lane 1), after 2 h, 4 h, and overnight (lanes 2 and 4, respectively) and detected with an anti-GST antibody. (**b**) The recombinant AHP2-GST, purified by GST-affinity chromatography, migrates on the SDS gel with an apparent molecular mass of 44 kDa corresponding to the estimated theoretical molecular mass. Protein bands were visualized by colloidal Coomassie staining. (**c**) GST-tagged AHP2 was purified by GST-affinity chromatography, separated by SDS-PAGE and visualized by immunoblotting using an anti-GST antibody.

The AHK1 phosphorelay downstream binding partner AHP2 identified in previous studies [35,36] was overexpressed as GST-tagged fusion protein in *E. coli* BL21 (DE3)-Gold cells overnight at 16 °C (Figure 5a) and purified to apparent homogeneity from bacterial extracts using glutathione-S-transferase sepharose (GE Healthcare). A single band on SDS–PAGE confirmed the homogeneity of the recombinant protein (Figure 5b). Identity of AHP2-GST was confirmed with anti-GST antibody (Figure 5c).

### 2.6. Interaction Studies of AHK1 Sensor Kinase with AHP2 Phosphotransfer Protein

**Figure 6:**
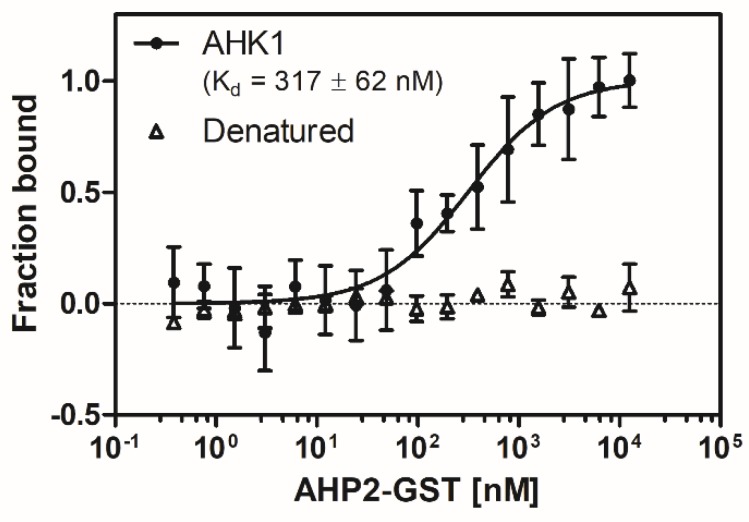
Microscale thermophoresis (MST) interaction studies of fluorescently-labeled AHK1 sensor kinase with GST-fusion of AHP2. Relative changes observed thermophoresis reflecting binding of the two protein partners (●) at different concentrations of AHP2-GST were fitted according to a one-binding-site model. From the related binding curve a K_d_ value of 317 +/− 62 nM was obtained for the AHK1-AHP2 complex formation. Data for chemically denatured AHK1 (△) which show no changes in thermophoresis are given for comparison. Data represent the mean of seven independent measurements +/− standard deviation. For control with denatured protein duplicates were taken for each concentration.

MST was used to monitor and quantify binding of histidine phosphotransfer protein AHP2 to fluorescently labeled full-length AHK1 sensor kinase. Changes observed in thermophoresis at different concentrations of AHP2-GST added to 40 nM AHK1 labeled with fluorescent dye AlexaFluor488-NHS were plotted as function of the AHP2 concentrations (0.4 nM–12.5 µM) used in the titration series (see Figure 6). The related binding curve corresponds to an apparent dissociation constant (K_d_) of 317 +/− 62 nM. This relatively low value provides evidence of a high affinity interaction between AHK1 and AHP2 and further supports functional folding of the purified recombinant full-length sensor kinase. Notably, a negative control using chemically denatured AHK1 shows no interaction with AHP2. 

### 2.7. AHK1 Acts as Phospho-His and/or Phospho-Asp Phosphatase In Vitro

**Figure 7:**
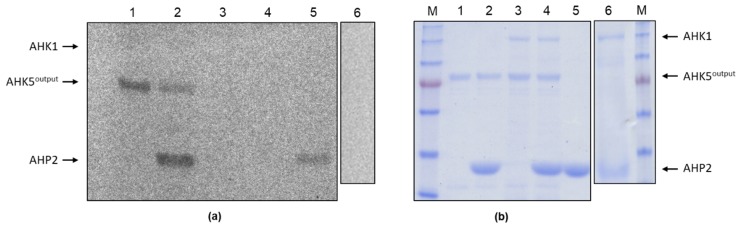
AHK1 functions as phospho-His and/or phospho-Asp phosphatase in vitro. (**a**) Autoradiogram of the phosphorylation experiments performed with (His)6-tagged AHK1 (AHK1), (His)6-tagged AHK5output (AHK5output) and GST-tagged AHP2 (AHP2) in the presence of γ-^33^P-ATP. Lane 1: AHK5output; lane 2: AHK5output and AHP2; lane 3: AHK1, AHK5output; lane 4: AHK1, AHK5output, AHP2; lane 5: AHP2; lane 6: AHK1, AHP2. Phosphorylation reactions were run at room temperature for 20 min. Thereafter, the proteins were separated by SDS-PAGE and the gel exposed to a phospho-imaging plate overnight. (**b**) Coomassie-stained SDS-PAGE gel showing the presence of the recombinant proteins in the reactions indicated in (a). M: protein size marker.

To test whether AHK1 has enzymatic activity, we performed auto-phosphorylation assays using γ-^33^P-ATP. However, no auto-modification of AHK1 with ^33^phosphate was observed. This suggested that AHK1 is not an active His kinase in the absence of the yet unknown ligand. However, as reported for the cytokinin receptor AHK4 [37], plant histidine kinases can also act as P-His or P-Asp phosphatases by forcing a backward flux of phosphoryl residues within a given phosphorelay system. To establish such an phosphorelay system in vitro, we first tested the capacity of the (His)_6_-tagged output domain of the soluble *Arabidopsis thaliana* histidine kinase 5 (AHK5^output^; amino acid 343 to 922), which contains the His kinase-typical HisKA, HATPAse c and the receiver domains [21] to auto-phosphorylate and to transfer the phosphoryl group to GST-tagged AHP2. When incubated with γ-^33^P-ATP, AHK5^output^ clearly displayed incorporation of ^33^phosphate (Figure 7, lane 1), demonstrating its auto-phosphorylation capacity. When GST-tagged AHP2 was present in the phosphorylation reaction, AHK5^output^ was able to transfer ^33^P-labeled phosphoryl residues to the phosphotransfer protein, as shown by the enhanced labelling of AHP2 against background and, in parallel, by the reduction of AHK5^output^ auto-labelling (Figure 7, compare lanes 2 and 5). In the presence of AHK1, however, neither auto-phosphorylation of AHK5^output^ (Figure 7, lane 3) nor the background-caused and AHK5^output^-caused enhanced labelling of AHP2 (Figure 7, lanes 4 and 6) were observed anymore. These results indicate that AHK1 is able to empty the AHK5^output^-to-AHP2 phosphorelay system from phosphoryl residues and, therefore, acts as P-His and/or P-Asp phosphatase in vitro.

## 3. Discussion

Despite the large number of histidine kinases participating in two-component signal transfer processes and their counterparts in plants involved in multi-step phosphorelay signaling, our information on thermodynamics and kinetics that determine the interaction of these sensor kinases with their downstream histidine phosphotransfer proteins as well as the conformational changes triggering signal transfer activity in the phoshorelay is still sparse. This is partly due to the fact that most of these sensor kinases are integral membrane proteins which are hardly accessible to biochemical characterization. Thus, not surprisingly, most of the published interaction studies are based on yeast-two-hybrid analysis allowing qualitative assessments on the interaction, although two systems have been addressed in a quantitative way in biophysical studies by fluorescence polarization [23] assay or surface plasmon resonance (SPR) experiments [38] in the past.

Among the five receptor histidine kinases described in *Arabidopsis*, AHK1 takes a special place for its potential involvement in osmoregulation and its role as a positive regulator in drought response [39], although the ligand of this receptor has remained unknown so far. In this study, to the best of our knowledge, AHK1 has been successfully produced heterologously in the enterobacterium *E. coli* in a functional state for the first time. Successful expression in a bacterial host turned out to be critically dependent on the presence of rare tRNAs. Furthermore, expression at a low temperature (16 °C), short expression time (5 h) and the addition of 2% (*v*/*v*) of ethanol were proven to be crucial for a successful expression of the full-length integral membrane protein. The critical importance of these parameters for a successful recombinant production of the sensor kinase probably lies in the fact that higher expression level will result in intracellular protein aggregation, non-functional protein, or negative interference with bacterial TCSs. Hence, in order to obtain the recombinant sensor kinase in a functional state for further physiological studies, strict monitoring of the heterologous expression is crucial. Solubilization and purification of AHK1 from the bacterial membranes was possible with the lipid-like detergent FosCholine-16 at high protein purity and yield leaving only minor background contaminants in the sample purified by single-step affinity chromatography. In order to confirm the high purity and monodispersity of the AHK1 produced, the solubilized and purified membrane protein was subjected to gel filtration. The resulting SEC profile confirms that only minor contaminants were present, which due to fact that they elute at a lower elution volume, might represent aggregates, larger oligomers of solubilized AHK1, or larger micelle-protein complexes. The apparent molecular weight of 111 kDa, calculated for the protein eluting at the main peak is somewhat lower than the sequence-based molecular mass expected for the full-length AHK1 sensor kinase. The deviation from the theoretical molecular mass might be related to detergent effects on the column matrix or a non-globular fold of the purified membrane receptor. Functional folding of recombinant AHK1 was verified by CD spectroscopy and by its ability to interact *in vitro* with the downstream phosphorelay element AHP2 [36]. Still, the apparent size observed for the purified recombinant AHK1 suggests that the protein elutes in a monomeric state. At first glance, this result appeared to be in disagreement with the dimeric or higher oligomeric states observed for other plant histidine kinases such as the receptors of the plant hormones ethylene and cytokinin [40]. Nonetheless, monomeric states of sensor histidine kinases have been also observed in solution and crystals in the past, as in the case of chemoreceptor Tlp1 from *Campylobacter jejuni* [41].

Remarkably, despite the apparent monomeric state under size exclusion chromatography conditions, the functionality of AHK1 in terms of potential to form homodimers could be shown in MST. The homomeric affinities of the AHK1 osmosensor strongly resemble the affinities observed for other sensor histidine kinases like AtETR1 and AtETR2 [7]. The same experimental setup and method, under which a dissociation constant of 208 nM was observed in this study for AHK1, showed values of 326 nM and 96 nM for AtETR1 and AtETR2 respectively [7]. The *in vitro* MST results of AHK1 homomerization were confirmed by two independent *in vivo* approaches, namely yeast mbSUS and in planta FRET-FLIM. The latter technique, in combination with confocal microscopy, demonstrates that AHK1 homomerization takes places in the plasma membrane of plant cells.

MST additionally revealed a high affinity interaction of AHK1 with AHP2. The dissociation constant of 300 nM for the AHK1-AHP2 interaction is in the same K_d_ range as observed for the interaction of other histidine kinases with their cognate histidine-containing phosphotransfer proteins (HPs). Interaction of the ethylene receptor ETR1 and the histidine phosphotransfer protein AHP1 is characterized by a K_d_ of 1.5 µM [23], affinities for sensor kinase AHK5 with its three interactors AHP1, AHP2, and AHP3 are in the range of 2.7 to 4.4 µM [38]. Still, the affinity of AHK1 to AHP2 is the tightest interaction observed for this type of signaling system known to date indicating strong interaction and high stability of the related phosphorelay complex.

The purified AHK1 protein also enabled us to perform *in vitro* auto-phosphorylation assays. However, no auto-phosphorylation was observed which we attribute to the missing AHK1 ligand in the phosphorylation reactions. However, this did not exclude the possibility that ligand-less AHK1 may act as P-His and/or P-Asp phosphatase, as it was, for instance, described for the cytokinin receptor AHK4 in the absence of the hormonal ligand [37]. To test this possibility, we successfully established an *in vitro* phosphorelay system consisting of the active output domain of soluble AHK5 (AHK5^output^) and AHP2. AHK5^output^ was not only able to auto-phosphorylate but also to transfer phosphoryl residues onto AHP2. Notably, AHP2 showed background labelling in the absence of AHK5^output^, which we attribute to the activity of an *E. coli* His kinase contaminating the AHP2 sample. However, in the presence of AHK1, the ^33^P-auto-labelling of AHK5^output^ and ^33^P-labelling of AHP2 were no longer detectable. This observation can be explainable by an AHK1-intrinsic phospho-His and/or phospho-Asp phosphatase activity, which, on the one hand, relays the phosphoryl residues directly from AHK5^output^, and on the other hand, is also able to relay phosphoryl residues from phosphorylated AHP2 on itself. Finally, AHK1 must lose its phosphoryl residues rapidly with a short halftime, eventually emptying the γ-^33^P-ATP pool in the reaction mixture. Interestingly, we were never able to observe a heteromer formation of full length AHK5 and AHK5^output^ with AHK1 via mbSUS and FRET-FLIM. However, the association of kinases and phosphatases with their target substrates can be very transient, impeding the detection of interaction by these and other (e.g., biochemical) approaches. As AHK1 interacts with AHP2 strongly, it is very likely that AHK1 preferentially relays the phosphoryl residues from AHP2 on itself and not from AHK5^output^ directly. However, we cannot entirely exclude the possibility that AHK1 also competes with AHK5^output^ for the availability of γ-^33^P-ATP directly.

With the purification protocol of AHK1 presented in this work a tool is provided to gain further in-depth knowledge on phosphorelay histidine kinases and their HPs, their interactions and physiological relevance. Moreover, with the in vitro AHK1-(AHK5^output^)-AHP2 phosphorelay system it now seems possible to determine the molecular mechanism of AHK1 sensing and the identity of the signaling molecule triggering AHK1 activity in further biochemical studies. Naturally, structural studies on the isolated AHK1 or the AHK1-AHP2 complex are possible, too. Here, the high purity and monodispersity of AHK1 opens systematic crystallization studies of the receptor, hopefully resulting in the first high-resolution structure of an integral membrane plant receptor histidine kinase.

## 4. Materials and Methods 

### 4.1. Cloning

*AHK1* was cloned into the pET16b-H10-TEV vector for expression using Gibson assembly [42]. The cDNA coding for AHP2 was cloned into a pGEX-6P-1 vector to generate a GST-fusion construct. The cDNA encoding AHK5^output^ was cloned into the pET-DEST42 vector to generate a C-terminal (His)_6_ fusion construct.

The following oligonucleotides were used in the process:
AHK5^output^-F5‘-GGGGACAAGTTTGTACAAAAAAGCAGGCT gtatggataacgctgtgagaaaggc-3‘AHK5^output^-R5′-GGGGACGACTTTGTACAAGAAAGCTGGGT tgtgcaaatactgttgcaaacactc-3′ ColE1-F5′-ggagcgaacgacctacaccgaactgagatacctacagcg-3‘gib_pET16b-TEV-rev5′-ATGTCCCTGAAAATACAG-3‘gib-pET16b-AHK1-for5′-ctgtattttcagggacatATGCGAGGAGATAGCTTC-3‘gib-pET16b-AHK1-rev5′-gttagcagccggatccttaAGCGGACAATGAAGTTTG-3‘gib_pET16b-TEV-for5′-TAAGGATCCGGCTGCTAAC-3‘ColE1-R5′-cgctgtaggtatctcagttcggtgtaggtcgttcgctcc -3‘

### 4.2. Expression of Recombinant Proteins

For the expression of AHK1 the *E. coli* strain BL21 (DE3), containing the pRARE plasmid (Novagen, Darmstadt, Germany), which encodes for rare tRNAs, was used. The overnight pre-culture was inoculated with a colony picked from a 1.5% agar plate and grown in 2YT medium (5 g/L NaCl, 10 g/L yeast extract, and 16 g/L peptone) containing ampicillin (100 µg/mL) and chloramphenicol (34 µg/mL) as selection markers, at 37 °C and 180 rpm. The expression culture was inoculated to an optical density (OD) of 0.15 measured at 600 nm in Terrific broth (TB) medium (5 g/L glycerol, 24 g/L yeast extract, 12 g/L peptone, 1.82 g/L KH_2_PO_4_, 19.76 g/L K_2_HPO_4,_ and 100 µg/mL ampicillin) containing 2% (*v*/*v*) ethanol. At an OD_600_ of 0.3–0.35 at 37 °C, cultures were cooled down to 30 °C until they reach an optical density of 0.4–0.45, where they were cooled down to their final expression temperature of 16 °C. The induction with 0.5 mM isopropyl β-d-1-thiogalactopyranoside (IPTG) was performed at an OD_600_ of 0.6. Following an incubation at 16 °C and 180 rpm, the cells were harvested after 5 h at 7500 × g and 4 °C for 15 min. The cell pellets were then frozen in liquid nitrogen and stored at −20 °C. In contrast to AHK1, AHP2 was transformed into BL21 (DE3) Gold cells, expressed in 2YT medium containing ampicillin (100 µg/mL) and 2% (*v*/*v*) ethanol. After reaching an OD_600_ of 0.4–0.5 the cultures were cooled down to 16 °C and induced at an OD_600_ of 0.8 with 0.1 mM IPTG. The cells were grown overnight and harvested as described above.

For the expression of AHK5^output^ the *E. coli* strain BL21 (DE3) was grown using Terrific broth (TB) medium. The LB overnight culture was diluted with TB medium to an OD_600nm_ of approximately 0.4. The cells were further grown for approximately 4 h at 37 °C until they reached the logarithmic phase at an OD_600nm_ of 0.6. Protein expression was induced by addition of 0.5 mM IPTG and cells were incubated for 20 h at 20 °C and harvested as described above.

Expression success was analyzed by SDS-PAGE and semidry Western blotting and electrogenerated chemiluminescence (ECL) detection after immunostaining with the indicated antibodies.

### 4.3. Purification of Recombinant Proteins

During the whole purification, the samples were kept on ice or at 4 °C. The cell pellet of the membrane-bound protein AHK1 was resuspended in 5 mL lysis buffer L (50 mM Tris, 300 mM NaCl, 10% (*w*/*v*) glycerol, 5 mM DTT, 0.002% (*w*/*v*) phenylmethylsulfonyl fluoride (PMSF), DNaseI, pH 7.6) per g of cell pellet. In each 50 mL of lysis buffer one tablet of protease inhibitor (cOmplete ULTRA, EDTA-free, Roche, Basel, Switzerland) was added. The lysis was performed using a cell disruption system (Constant Systems) at 2.4 kbar. For first removal of unwanted cell debris and inclusion bodies, the lysate was centrifuged at 14,000 × g for 30 min. To collect the membrane fractions, the supernatant was centrifuged for another 30 min at 40,000 × g. After resuspension, equal distribution, and centrifugation at 34,000 × g for 30 min, the membrane pellets were frozen in liquid nitrogen and stored at −80 °C. AHK1 was solubilized in 1% (*w*/*v*) FosCholine-16 in buffer A (50 mM Tris, 300 mM NaCl, 10% (*w*/*v*) glycerol, 2.5 mM DTT, 0.002% (*w*/*v*) PMSF, pH 7.6) for 1 h at 700 rpm. After centrifugation at 230,000 × g for 30 min, another 0.002% (*w*/*v*) PMSF were added to the supernatant, which was loaded onto a 5 mL Ni-NTA HisTrap FF column (GE Healthcare, Chicago, IL, USA) using an ÄKTAPrime Plus system (GE Healthcare). Prior to loading, the column was equilibrated with at least 10 bed volumes of buffer A with 0.015% (*w*/*v*) FosCholine-16. A washing step was performed with buffer A containing 50 mM imidazole and elution followed with buffer A at 100 mM imidazole. After pooling and concentrating the samples by centrifugation with a 50 kDa Amicon Ultra-15 tube (Merck Millipore, Darmstadt, Germany), the buffer was exchanged using a PD 10 or PD MiniTrap G-25 desalting column (GE Healthcare).

The AHP2 cell pellets were resuspended with PBS buffer (140 mM NaCl, 2.7 mM KCl, 10 mM Na_2_HPO_4_, 1.8 mM KH_2_PO_4_, pH 7.3) with 0.002% (*w*/*v*) PMSF, lysed in two cycles using a pre-cooled French pressure cell (Thermo, Waltham, MA, USA) with approximately 1 kbar (14,000 psi). After ultracentrifugation at 230,000 × g for 1 h, the supernatant was loaded on an equilibrated GSTrap HP 5 mL column (GE Healthcare Life Sciences) using an ÄKTAPrime Plus system. Whereas equilibration and washing were performed with at least five bed volumes with the PBS buffer, the protein was released by competitive binding with elution buffer E (50 mM Tris, 10 mM reduced glutathione, pH 8.0). Concentrating (10 kDa cut-off) and buffer exchange to storage buffer (50 mM Tris, 300 mM NaCl, pH 7.6) was done as described. For storage, aliquots containing 20% (*w*/*v*) glycerol were shock-frozen and kept at −80 °C.

The AHK5^output^-(His)_6_ expressing *E. coli* cells were harvested by centrifugation at 4000 g for 5 min and resuspended in 10 mL pre-cooled NPI-10 buffer (50 mM NaH_2_PO_4_∙H_2_O, 300 mM NaCl, 10 mM imidazole, pH 8.0) containing a proteinase inhibitor cocktail (Bimake). Glass beads (diameter: 0.25–0.5 mm) were added and the cells were lysed by vortexing at 4 °C for 10 min. The lysate was centrifuged at 4 °C and 15000 × g for 40 min and the supernatant subjected to native protein purification using Ni-NTA Superflow Columns according to the manufacturer’s protocol (QIAGEN).

Purification was verified by SDS-PAGE and Western blotting.

### 4.4. Size Exclusion Chromatography

Gel filtration was performed on a 10/300 Superose 6 Increase column by GE Healthcare Life Sciences using an ÄKTAPrime Plus system. At a flow rate of 0.1 mL min^-1^, 100 µg of protein in a volume of 50 µL were applied to the column via a 100 µL loop. The system was equilibrated in buffer A with 0.015% (*w*/*v*) FosCholine-16 (see 4.3). Column calibration was performed as a four-point calibration with thyroglobuline (669 kDa, bovine thyroid), ferritin (440 kDa, horse spleen), aldolase (158 kDa, rabbit muscle), and ovalbumin (43 kDa, hen egg) according to the instructions from the Gel Filtration Calibration Kit HMW (GE Healthcare Life Sciences).

### 4.5. Circular Dichroism Spectroscopy

For CD spectroscopy the protein was rebuffered to the CD buffer (50 mM K_2_HPO_4_/KH_2_PO_4_, 0.015% (*w*/*v*) FosCholine-16, pH 7.6). Spectra were recorded with a protein concentration of 0.1 mg/mL in 200 µL in a JASCO J-810 CD spectropolarimeter at room temperature. In the wavelength range between 190 nm and 260 nm, 20 spectra were accumulated. Deconvolution was performed with the CDPRO suite [30]. SMP65 was used as a reference data set which included 65 membrane proteins. The SOPMA analysis was performed with the full-length sequence of H10-TEV-AHK1 [32].

### 4.6. Fluorescent Labeling of Purified Protein

To enable the measurement of binding studies using microscale thermophoresis, AHK1 was labeled with the fluorophore AlexaFluor488-NHS (Invitrogen, Thermo Fisher, Waltham, MA, USA). Therefore, the protein was incubated with 2.5 × the amount of the fluorophore in a total volume of 500 µL labeling buffer (50 mM K_2_HPO_4_/KH_2_PO_4_, 300 mM NaCl, 0.015% (*w*/*v*) FosCholine-16, pH 7.6) for 30 min in the dark during gentle agitation. The following buffer change to MST buffer 1 (50 mM Tris, 300 mM NaCl, 0.015% (*w*/*v*) FosCholine-16, pH 7.6) was performed using a PD Mini desalting column. After measuring the protein concentration and labeling efficiency, 20% (*w*/*v*) glycerol was added and aliquots for the MST measurement were frozen in liquid nitrogen.

### 4.7. Interaction Studies by MST, mbSUS, and FRET-FLIM Analyses

To verify the functionality of the purified proteins, the binding of AHK1 to AHP2 was measured. Therefore, the Monolith NT 115 and standard capillaries (NanoTemper Technologies, München, Germany) were used. Furthermore, the MST power was set to 40% and the LED power to 30%. The binding study was performed in seven measurements, whereas the negative control with denatured protein in a duplicate. The labeled AHK1 was diluted to a final measuring concentration of 40 nM using MST buffer 2 (50 mM HEPES, 300 mM NaCl, 0.015% (*w*/*v*) FosCholine-16.5% (*w*/*v*) glycerol, pH 7.6). The dilution series of AHP2 from 12.5 µM to 0.4 nM was done using MST buffer 3 (50 mM Tris, 300 mM NaCl, 5% (*w*/*v*) glycerol, pH 7.6). The labeled AHK1 was incubated with the non-labeled AHP2 in a ratio of 1:1 resulting in the prior concentrations. After an incubation of 5 min in the dark, the samples were loaded into the glass capillaries for the measurement. For the negative controls, a denaturing buffer D1 (4% (*w*/*v*) SDS, 80 mM DTT in MST buffer 2 containing only 0.0075% (*w*/*v*) FosCholine-16 in total) was added to the mixed proteins of interest. As evaluation strategy T-Jump was used.

Dimerization of AHK1 was analyzed by MST measurements, using 40% MST power and 70% LED power. The labeled AHK1 was treated as described before. The interacting unlabeled AHK1 was diluted in a series from 12 µM to 0.4 nM in MST buffer 4 (50 mM Tris, 300 mM NaCl, 0.015% (*w*/*v*) FosCholine-16, 20% (*w*/*v*) glycerol, pH 7.6). After mixing the samples in a ratio of 1:1 and incubation in the dark for 5 min, they were loaded into the standard capillaries and measured. For the triplicate of the negative control, denaturing buffer D2 (4% (*w*/*v*) SDS, 80 mM DTT, 156 mM NaCl, 26 mM Tris, 0.015% (*w*/*v*) FosCholine-16, 12.5% (*w*/*v*) glycerol, pH 7.6) was added in a ratio of 1:1 to the prior mixed interacting proteins. For binding analysis, initial fluorescence was used.

Yeast mbSUS studies were performed as described before [33]. For confocal imaging and FRET-FLIM analyses, full-length AHK1 was expressed as C-terminal GFP and C-terminal mCherry fusion in *Agrobacterium tumefaciens*-mediated transiently-transformed *Nicotiana benthamiana* epidermal leaf cells according to [34]. Confocal imaging and fluorescence life measurements were done as described before [34]. The statistical analysis was carried out with JMP 14 software available at https://www.jmp.com. The homogeneity of variance was tested with a Brown–Forsythe test. A non-parametric Kruskal–Wallis test was then performed followed by a Dunn’s post hoc test. The boxplot representation was designed with http://shiny.chemgrid.org/boxplotr/.

### 4.8. In Vitro Phosphorylation and Phosphorelay Assays

Approximately 3 µg of AHK1-(His)_6_, AHK5^output^-(His)_6_, and AHP2-GST were incubated at the indicated combinations (see Figure 7) with 1.1 µl γ-^33^P-ATP (3700 MBq/mL; Hartmann Analytics, Braunschweig, Germany) in a final volume of 30.1 µl TEDG buffer (50 mM Tris, 0.5 mM EDTA, 2 mM DTT, 50 mM KCl, 5 mM MgCl_2_, 10% glycerol, pH 8.0) at room temperature for 30 min. The reaction was stopped by addition of 5 µl of SDS-PAGE sample buffer. The samples were analyzed by SDS-PAGE and the gel was subsequently imaged overnight using a radiosensitive phospho-imaging plate. Detection was carried out using the BAS cassette of the Fluorescence Laser Imaging Scanner FLA-3000/300G according to the manufacturer´s instructions (FUJIFILM). A Coomassie-stained SDS-PAGE gel loaded in parallel with the same samples but in the absence of γ-^33^P-ATP was used as a loading control.

### 4.9. Analytical Methods 

Protein concentrations were determined by the bicinchoninic acid assay (Perbio Science, Bonn, Germany) for the AHK1 sensor kinase and by the Bio-Rad protein assay (Bio-Rad, München, Germany) for AHP2-GST using bovine serum albumin as a standard. Purification of recombinant proteins was examined by SDS-PAGE as described by Laemmli [43] and Schägger and Jagow [44]. Proteins were separated on 10–12% polyacrylamide gels and visualized by colloidal Coomassie staining according to Kang et al. [45,46].

## Figures and Tables

**Table 1:** The secondary structures of purified recombinant AHK1. Deconvolution results from CD spectra acquired with purified recombinant AHK1 by algorithms SELCON3, CONTINLL, and CDSSTR are shown together with its sequence-based in-silico secondary structures predicted by SOPMA.

Algorithm	% Helical Content	% Beta Strand Content	% Turn Content	% Unordered Content
**SELCON3**	26.8	20.0	21.9	27.4
**CONTINLL**	31.2	21.8	20.0	27.0
**CDSSTR**	34.9	20.4	17.7	26.7
**GOR4 prediction**	34.9	19.4	0	45.7
**SOPMA prediction**	39.4	15.5	3.9	41.3

## Supplementary Materials

The following are available online at https://www.mdpi.com/2223-7747/9/3/304/s1, Figure S1. Expression of His-tagged AHK1 in BL21 (DE3) *E. coli* cells containing the pRARE plasmid under different growth conditions. For analysis, the samples were separated on a 10% SDS-PAGE gel and identified after Western blotting with an anti-his-antibody; (**a**) The cells were grown in 2YT medium at 30 °C after induction with 0.5 mM IPTG. Samples were taken every hour and show a strong signal for possible degradation bands at around 35 kDa. (**b**) *E. coli* cells were grown in 2YT medium at 16 °C and an addition of 2% ethanol. Samples were taken every 2 h after induction with 0.5 mM IPTG and show only a faint band after 4 h. (**c**) The protein expression in TB medium at 16 °C and the additional 2% (*v*/*v*) ethanol was sampled after 0 h, 2 h, 4 h and 5 h following induction with 0.5 mM IPTG. Under these conditions the most prominent protein bands with the least degradation or incomplete translated proteins were detected; Figure S2. Detergent screening for solubilization of AHK1 in 100 µL. P resembles the pellet directly after resuspension in buffer A, which was subsequently incubated with the ×-fold critical micell concentration (cmc) of the stated detergent for 1 h at 4 °C and 700 rpm. Detergents were selected from the JBScreen Detergent set (Jena Bioscience). The pellet (P) and supernatant (SN) after centrifugation at 230,000 × g for 30 min were analyzed using SDS-PAGE and Western blotting; (**a**) The detergents C12E8, Cymal-6 and LMNG were used at a final concentration of 7.5 × cmc, whereas OG was used in a lower concentration of 3 × cmc and Fos12 at 4.5 × cmc. Fos12 is the only detergent showing a band of AHK1 in the supernatant fraction. (**b**) Whereas HTG was tested with the 3 × cmc, the other detergents ANAPOE-X-114, LDAO and Fos16 were tested with the 7.5 × cmc. Fos16 was not part of the JBScreen Detergent set and used in a comparable cmc to provide better comparison. Only LDAO shows a faint band in the supernatant identified as AHK1 and still a more prominent band in the pellet fraction, suggesting incomplete solubilization; Figure S3. Detergent screening for solubilization of AHK1 in larger volumes of 2 mL. P resembles the pellet directly after resuspension in buffer A, which was subsequently incubated with the ×-fold cmc of the stated detergent for 1 h at 4 °C and 700 rpm. The detergents were used with either 1% or 2% (*w*/*v*). The pellet (P) and supernatant (SN) after centrifugation at 230,000 × g for 30 min were analyzed using SDS-PAGE and Western blotting; (**a**) The detergents Na-cholate and CHAPS were used with 2% (*w*/*v*), whereas Fos16, DDM and Tween-20 with 1% (*w*/*v*). Fos16 is the only detergent showing a band of AHK1 in the supernatant fraction. (**b**) Whilst Fos12 and LDAO were tested with 1% (*w*/*v*), Fos10 due to its higher cmc was analyzed at a final concentration of 2% (*w*/*v*). Both FosCholines show a distinct band in the supernatant, speaking for its solubilization. LDAO also shows a more prominent band in the supernatant, but also one remaining in the pellet, suggesting still incomplete solubilization even at a higher concentration than before. Table S1. Analyzed detergents and their properties.
