# Supplementary material for: High-Level Expression, Purification and Initial Characterization of Recombinant Arabidopsis Histidine Kinase AHK1
Source: Plants (Basel). 2020 Mar 1;9(3):304. doi: 10.3390/plants9030304 (PMC7154865; doi:10.3390/plants9030304)
Supplement: Supplementary file 1 [file plants-09-00304-s001.pdf]

plants

MDPI

Supplement

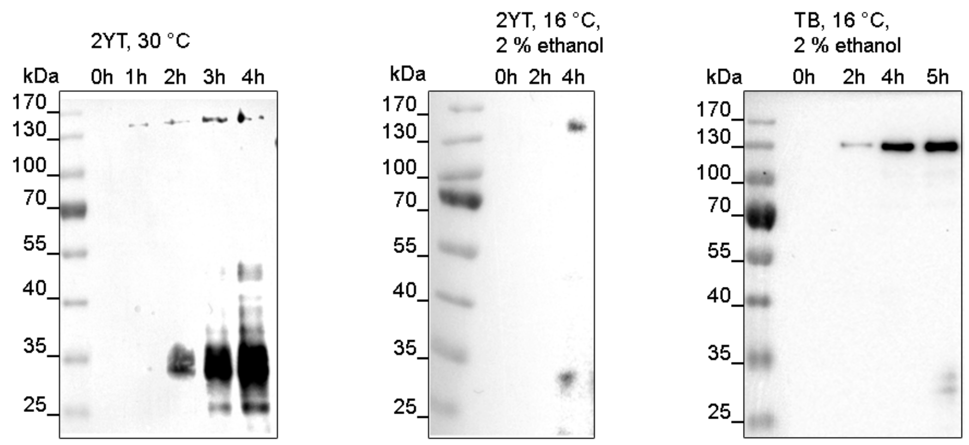

(**a**) (**b**) (**c**)

Figure S1. Expression of His-tagged AHK1 in BL21 (DE3) *E. coli* cells containing the pRARE plasmid under different growth conditions. For analysis, the samples were separated on a 10 % SDS-PAGE gel and identified after Western blotting with an anti-his-antibody; (**a**) The cells were grown in 2YT medium at 30 °C after induction with 0.5 mM IPTG. Samples were taken every hour and show a strong signal for possible degradation bands at around 35 kDa. (**b**) *E. coli* cells were grown in 2YT medium at 16 °C and an addition of 2 % ethanol. Samples were taken every 2 h after induction with 0.5 mM IPTG and show only a faint band after 4 h. (**c**) The protein expression in TB medium at 16 °C and the additional 2 % (v/v) ethanol was sampled after 0 h, 2 h, 4 h and 5 h following induction with 0.5 mM IPTG. Under these conditions the most prominent protein bands with the least degradation or incomplete translated proteins were detected.

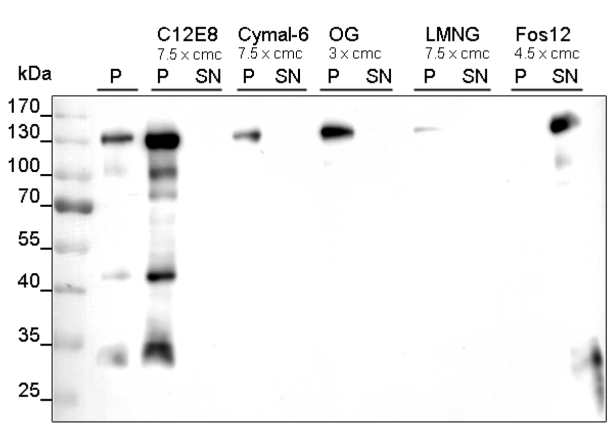

(**a**)

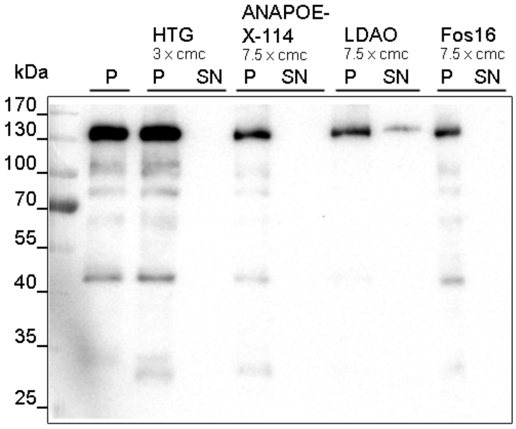

(**b**)

Figure S2. Detergent screening for solubilization of AHK1 in 100 µL. P resembles the pellet directly after resuspension in buffer A, which was subsequently incubated with the x-fold critical micell concentration (cmc) of the stated detergent for 1 h at 4 °C and 700 rpm. Detergents were selected from the JBScreen Detergent set (Jena Bioscience). The pellet (P) and supernatant (SN) after centrifugation at 230,000 x g for 30 min were analyzed using SDS-PAGE and Western blotting; (**a**) The detergents C12E8, Cymal-6 and LMNG were used at a final concentration of 7.5 x cmc, whereas OG was used in a lower concentration of 3 x cmc and Fos12 at 4.5 x cmc. Fos12 is the only detergent showing a band of AHK1 in the supernatant fraction. (**b**) Whereas HTG was tested with the 3 x cmc, the other detergents ANAPOE-X-114, LDAO and Fos16 were tested with the 7.5 x cmc. Fos16 was not part of the JBScreen Detergent set and used in a comparable cmc to provide better comparison. Only LDAO shows a faint band in the supernatant identified as AHK1 and still a more prominent band in the pellet fraction, suggesting incomplete solubilization.

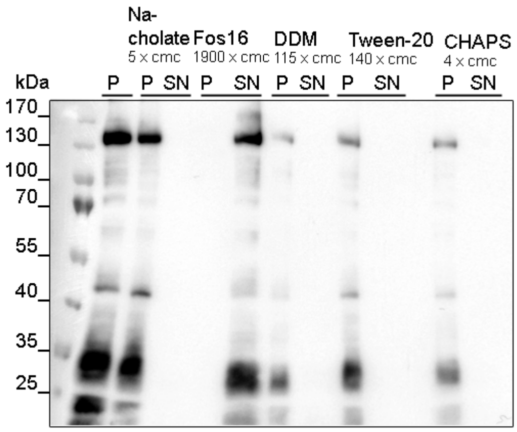

(**a**)

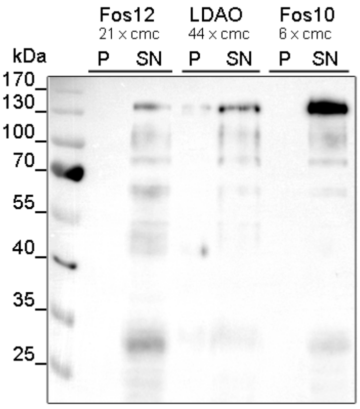

(**b**)

Figure S3. Detergent screening for solubilization of AHK1 in larger volumes of 2 mL. P resembles the pellet directly after resuspension in buffer A, which was subsequently incubated with the x-fold cmc of the stated detergent for 1 h at 4 °C and 700 rpm. The detergents were used with either 1 % or 2 % (w/v). The pellet (P) and supernatant (SN) after centrifugation at 230,000 x g for 30 min were analyzed using SDS-PAGE and Western blotting; (**a**) The detergents Na-cholate and CHAPS were used with 2 % (w/v), whereas Fos16, DDM and Tween-20 with 1 % (w/v). Fos16 is the only detergent showing a band of AHK1 in the supernatant fraction. (**b**) Whilst Fos12 and LDAO were tested with 1 % (w/v), Fos10 due to its higher cmc was analyzed at a final concentration of 2 % (w/v). Both FosCholines show a distinct band in the supernatant, speaking for its solubilization. LDAO also shows a more prominent band in the supernatant, but also one remaining in the pellet, suggesting still incomplete solubilization even at a higher concentration than before.

Table S1. Analyzed detergents and their properties.

Detergent	Full name	Type	cmc [mM]	cmc [% (w/v)]	Analyzed concentration [x cmc]
C12E8	-	Non ionic	0.09	0.005	7.5
Cymal-6		Non ionic	0.56	0.028	7.5
OG	n-Octyl-ß-D-glucoside	Non ionic	18	0.53	3
LMNG	Lauryl Maltose Neopentyl Glycol	Non ionic	0.01	0.001	7.5
Fos12	FosCholine®-12	Zwitterionic	1.5	0.047	4.5/21
HTG	n-Heptyl-ß-D-thioglucoside	Non ionic	29	0.85	3
ANAPOE®-X-114	-	Non ionic	0.2	0.011	7.5
LDAO	n-Dodecyl-N,N-dimethylamine-N-oxide	Zwitterionic	2	0.023	7.5/44
Fos16	FosCholine-16	Zwitterionic	0.013	0.00053	7.5/1900
Na-cholate	Sodium-cholate	Zwitterionic	9.5	0.41	5
DDM	n-dodecyl-ß-D-maltoside	Non ionic	0.17	0.0087	115
Tween-20	-	Non ionic	0.059	0.0072	140
CHAPS	3-[(3-cholamidopropyl)dimethylammonio]-1-propanesulfonate	Zwitterionic	8	0.49	4
Fos10	FosCholine-10	Zwitterionic	11	0.35	6

© 2020 by the authors. Licensee MDPI, Basel, Switzerland. This article is an open access article distributed under the terms and conditions of the Creative Commons Attribution (CC BY) license (http://creativecommons.org/licenses/by/4.0/).
